# Prevalence of sexually transmitted infections among pregnant women attending antenatal clinics in West Coast Region of The Gambia

**DOI:** 10.4314/ahs.v21i2.13

**Published:** 2021-06

**Authors:** Alphonsus Isara, Aru-Kumba Baldeh

**Affiliations:** 1 Department of Community Health, University of Benin, Benin City, Nigeria; 2 Vaccine and Immunity Unit, Medical Research Council, The Gambia

**Keywords:** Sexually transmitted infections, pregnant women, antenatal clinics, The Gambia

## Abstract

**Background:**

Sexually Transmitted Infections (STI) are the second most common cause of healthy life years lost by women in the 15 – 44 years age group in Africa.

**Aim/Objective:**

To determine the prevalence of STIs among pregnant women attending antenatal care (ANC) clinics in the West Coast Region of The Gambia.

**Materials and Methods:**

Blood, urine, and high vaginal swabs samples from 280 pregnant women attending ANC in Brikama District Hospital, Brikama, and Bandung Maternity and Child Health Hospital, Bandung were examined. Serum samples were tested for HIV using western blot technique and for syphilis using the Venereal Disease Research Laboratory (VDRL) test, and rapid plasma regimen. Candida albicans, Group B Streptococcus and Neisseria gonorrhoea were identified using Analytical Profile Index (API). Direct urine microscopy was used to identify C. albicans and Trichomonas vaginalis while Chlamydia trachomatis was identified using Direct Fluorescent Antibody (DFA) test.

**Results:**

The overall prevalence of STIs was 53.6%. The pathogenic agents isolated were Candida albicans (31.8%), Streptococcus agalactiae (15.0%), Treponema pallidum (6.8%), HIV (5.7%), Trichomonas vaginalis (3.9%), Neisseria gonorrhoea (1.8%) and Chlamydia trachomatis (0.7%). STIs were more prevalent among women in the younger age group of 15 – 24 years (54.7%), unemployed (54.0%), Primipara (62.3%), and in the third trimester of pregnancy (72.7%).

**Conclusion:**

A high prevalence of STIs was found among pregnant women attending ANC in the West Coast region of The Gambia. Public health intervention programmes should be strengthened to promote the sexual and reproductive health of pregnant women in The Gambia.

## Introduction

The burden of morbidity and mortality worldwide resulting from sexually transmitted pathogens compromises the quality of life, as well as sexual and reproductive health.^1^ Sexually Transmitted Infections (STIs) impose a substantial strain on the budgets of both households and national health systems in middle and low-income countries, and have an adverse effect on the overall well-being of individuals.^1^ The World Bank estimates indicate that STIs, excluding Human Immunodeficiency Virus (HIV), are the second most common cause of healthy life years lost by women in the 15 – 44 years age group in Africa and accounts for approximately 17% of the total burden of disease.^2^ The high prevalence of STIs has contributed to the disproportionately high HIV incidence and prevalence in many African countries including The Gambia. According to the World Health Organization (WHO) Global Health Risks Report, approximately one million women in Africa die yearly due to infection with HIV, Human Papilloma Virus (HPV), and other STIs.^3^

More than 30 different bacteria, viruses, and parasites are known to be transmitted through sexual contact. Eight of these pathogens are linked to the greatest incidence of STIs.^4^ Of these eight infections, four are currently curable: syphilis, gonorrhoea, chlamydia, and trichomoniasis. The other four are viral infections which are incurable: hepatitis B, Herpes Simplex Virus (HSV), HIV, and HPV.^4^ STIs during pregnancy can have serious reproductive health consequences beyond the immediate impact of the infection itself e.g., infertility and mother-to-child transmission which can result in ectopic pregnancy, miscarriage, preterm labour and delivery, stillbirth, neonatal death, low-birth-weight and prematurity, sepsis, pneumonia, neonatal conjunctivitis, and congenital deformities.^4^,^5^ Long term morbidities such as cervical cancers, chronic hepatitis, and chronic pelvic infection have also been linked with STIs.^5^

There is a high prevalence of STIs in many developing countries (The Gambia is not an exception) and these continue to have an impact on pregnancy outcomes. Unfortunately, most of these countries rely on the syndromic approach to manage STIs in pregnancy - an approach that is poor in identifying asymptomatic infections.^6^

In The Gambia, the prevalence of STIs in pregnant women has not been extensively studied. Over three decades ago, a study on STIs amongst one hundred randomly selected ANC attendees in Bakau, The Gambia, revealed that the prevalence of infection with Candida albicans was 35%, Trichomonas vaginalis 32%, Chlamydia trachomatis 6.9%, Neisseria gonorrhoeae 6.7% and Treponema pallidum 1%.^7^ Since then, estimates of STIs amongst pregnant women have relied on sentinel studies among women of reproductive age and other key populations such as female sex workers. However, a study on STIs focusing on the general population showed that 28% of women and 5% of men were HSV2 ELISA positive while 10% of women and 2% of men were positive for syphilis.^8^ Another study in the general population revealed that the prevalence of HIV was 6.7%, while that of hepatitis C (HCV) was 2.1%, with both infections occurring more frequently in males than in females.^9^ In order to ascertain the current trends of STIs among pregnant women, this study was carried out to determine the prevalence of STIs among pregnant women attending ANC clinics in the West Coast Region of The Gambia.

## Materials and Methods

### Study area and settings

This study was carried out among pregnant women attending ANC clinics in Brikama District Hospital, Brikama, and Bandung Maternity and Child Health Hospital (formally Jammeh Foundation for Peace Hospital), Bandung, all in the West Coast Region of The Gambia between June and October 2017.

### Study population

The study included booked pregnant women attending antenatal clinics at the Jammeh Foundation for Peace Hospital and Brikama District Hospital which were located in the West Coast region of The Gambia. Pregnant women who were too ill to participate were excluded from the study.

### Recruitment of participants

Pregnant women who met the inclusion criteria were recruited consecutively for the study from the two hospitals during antenatal visits.

### Sample size calculation

The minimum sample size required for the study which was calculated using the formula for single proportions (n= zpq2/d2) was 273.

### Data collection

Data collection was by means of a questionnaire and specimen analysis. The questionnaire was used to collect socio-demographic and obstetric information from the pregnant women. Blood, urine and high vaginal swabs were the specimen analyzed.

### Specimen collection

Collection, transportation, and processing of samples were done according to the standard operating procedures. Urine samples were collected using a sterile urine container. High Vaginal Swabs (HVS) were collected using sterile gloves. A drop of normal saline was put on one frosted glass slide and the HVS was smeared on the frosted glass slide and covered with a cover slip. Three to 5mls of blood was drawn aseptically from each pregnant woman and was emptied into an Ethylenediamineetraacetic Acid (EDTA) vacutainer.

### Specimen analysis

Blood antibodies serological tests: HIV antibody assay was carried out using HIV 1 and 2 rapid test strips (Abbott laboratories-USA) and the positive samples were confirmed using the western blot method. For the detection of Treponema pallidum (syphilis), Venereal Disease Research Laboratory (VDRL) test and rapid plasma regimen were used to determine if the respondents had syphilis.

### Urine analysis

Wet preparation for the identification of parasites was performed by putting a drop of the centrifuged urine on a clean slide and the slide was covered with a cover slip and viewed under the microscope using X10, X20 and X40 magnifications for the detection of *Trichomonas vaginalis* and *Candida albicans*. The identification of Chlamydia trachomatis was done using Direct Fluorescent Antibody (DFA) test.

### High vaginal swab analysis

**Identification of *Neisseria gonorrhoea*:** The HVS samples were inoculated in Thayer Martin media and incubated for 18 – 48 hours in a carbon dioxide (CO2) incubator. Suspected colonies of Neisseria gonorrhoea were then isolated on chocolate agar. Oxidase test was carried out on the suspected colonies using Oxidase strip. Gram negative diplococcic were further tested using API NH for the identification of Neisseria gonorrhoea.

**Identification of *Candida albicans*:** HVS samples were inoculated into Sabouraud dextrose media and incubated for 18 – 48 hours. Analytical Profile Index (API) was used for the identification of Candida albicans.

**Identification of Group B *Streptococcus* (GBS):** HVS samples were inoculated on gentamicin blood agar and incubated for 18 – 24 hours. The suspected colonies of GBS were then cultured on blood agar to obtain pure colonies. Streptex rapid latex agglutination test and API for streptococcus was used for the identification of GBS.

### Statistical analysis

An initial univariate analysis was carried out for all the variables. The association between socio-demographic characteristics of the pregnant women and the prevalence of STIs was tested using Chi-square statistical test. Binary logistic regression was modeled to identify significant independent predictors of the prevalence of STIs. The level of significance was set at p < 0.05.

### Ethical consideration

Ethical approval was obtained from the Joint Gambia Government and Medical Research Council (MRC) Ethics Committee (protocol number R017 039). A written informed consent was obtained from each of the pregnant women before samples were collected from them. The participants were fully assured of confidentiality and anonymity before data collection.

## Results

A total of 280 consenting pregnant women aged 15 to 44 years participated in the study. A higher proportion of them 132 (47.2%) were in the age group 25 to 34 years. The median (inter-quartile range) age of the respondents was 25.5 (22.0 – 31.0) years. Almost all the respondents were married 276 (98.6%), Muslim 276 (98.6%) and not employed [276 (98.6%)]. A little more than half had no formal education 148 (52.9%), while 168 (60.0%) were of the Mandinka tribe. One hundred and twenty-six (45.0%) were grand-multipara, 168 (67.2%) had 1 to 4 surviving children and 161 (64.4%) were at the second trimester.

Table 1 shows the prevalence of STIs and the isolated pathogenic agents. The overall prevalence of STIs was 53.6%. The most prevalent pathogenic agent isolated was *Candida albicans* 89 (31.8%) followed by *Streptococcus agalactiae* 42 (15.0%), *Treponema pallidum* 19 (6.8%), HIV 16 (5.7%), *Trichomonas vaginalis* 11 (3.9%), *Neisseria gonorrhoea* 5 (1.8%), and the least prevalent was *Chlamydia trachomatis* 2(0.7%).

**Table 1:** Prevalence of Sexually Transmitted Infections and the isolated pathogenic agents

STIs	Frequency (n = 280)	Percent
**STI test results**		
Positive	150	53.6
Negative	130	46.4
**Pathogenic agent** *		
*Candida albicans*	89	31.8
*Streptococcus agalactiae*	42	15.0
*Treponema pallidum*	19	6.8
*Human Immunodeficiency Virus*	16	5.7
*Trichomonas vaginalis*	11	3.9
*Neisseria gonorrhoea*	5	1.8
*Chlamydia trachomatis*	2	0.7

* Multiple responses

The socio-demographic characteristics and prevalence of STIs among the pregnant women is shown in table 2. The proportion of pregnant women who tested positive for STIs reduced with increasing age group, 65 (57.2%) in the age group 15 – 24 years and 13 (38.2%) in the age group 35 – 44 years. The association between age group and STIs was not statistically significant (p=0.149). Similarly, all other socio-demographic variables did not show any statistical association with the development of STIs by the pregnant women. However, it was observed that all the respondents who were not married 4 (100.0%) were positive for STIs when compared to 146 (52.7%) who were married (p = 0.126). A higher proportion of Muslim 149 (54.0%) respondents and those who were not employed 149 (54.0%) tested positive for STIs. Considering parity, the prevalence of STIs showed a decreasing trend from primipara 38 (62.3%) through multipara 50 (53.8% to grand multiparity 62 (49.2%) [p = 0.241]. Thirty-five (61.4%) of the respondents with no child tested positive to STIs compared with 97 (50.8%) of those with 1–4 children and 18 (56.3%) of those with more than four children (p=0.351). A higher proportion of respondents had STIs in the third trimester 8 (72.7%) when compared with first 45 (53.6%) and second 97 (52.4%) trimesters (p=0.423).

**Table 2:** Socio-demographic characteristics and Prevalence of Sexually Transmitted Infections among the respondents

Variables	Prevalence of STIs		
		
	Positive [n=150 (%)]	Negative [n=130 (%)]	p-value
**Age group(Years)**			
15–24	65(57.0)	49(43.0)	0.149
25–34	72(54.5)	60(45.5)	
35–44	13(38.2)	21(61.8)	
**Marital Status**			
Married	146 (52.9)	130 (47.1)	0.126
Not married	4 (100.0)	0 (0.0)	
**Ethnicity**			
Wolof	16(55.2)	13(44.8)	0.988
Mandinka	89(53.0)	79(47.0)	
Fula	25(55.6)	20(44.4)	
Jola	12(50.0)	12(50.0)	
Others**	8 (57.1)	6(42.9)	
**Level of education**			
None	78 (53.4)	68 (46.6)	0.803
Primary	25 (55.6)	20 (44.4)	
Secondary	45 (54.2)	38 (45.8)	
Tertiary	2 (33.3)	4 (66.7)	
**Religion**			
Christianity	1(25.0)	3(75.0)	0.340
Islam	149(54.0)	127(46.0)	
**Employment status**			
Employed	1(25.0)	3(75.0)	0.340
Not employed	149(54.0)	127(46.0)	
**Parity**			
Primipara	38(62.3)	23(37.7)	0.241
Multipara	50(53.8)	43(46.2)	
Grandmultipara	62(49.2)	64(50.8)	
**Number of children**			
0	35 (61.4)	22 (38.6)	0.351
1–4	97 (50.8)	94 (49.2)	
>4	18 (56.3)	14 (43.80	
**Gestational Age**			
First trimester	45(53.6)	39(46.4)	0.423
Second trimester	97(52.4)	88(47.6)	
Third trimester	8(72.7)	3(27.3)	

When all the socio-demographic variables were fitted into the binary logistic regression model to identify independent predictors of STIs among the pregnant women; it was found that marital status and parity could be possible predictors of STIs. Married respondents were four times likely to have STIs when compared with their counterparts who were not married (AOR 3.66, 95% CI 0.35 – 37.84). In a similar way, primiparous respondents were four times likely to have STIs when compared with those who are grand multiparous (AOR 3.86, 95% CI 0.37 – 40.33) [table 3].

**Table 3:** Independent predictors of Sexually Transmitted Diseases among the pregnant women

Variables	Regression coefficient	p-value	AOR (95% CI)
**Age (Years)**	-0.05	0.139	0.95 (0.90 – 1.02)
**Marital Status**			
Married	1.297	0.267	3.66 (0.35 – 37.84)
Not married*			1
**Ethnicity**			
Wolof*			
Mandinka	-0.196	0.639	0.82 (0.36 – 1.87)
Fula	-0.084	0.864	0.92 (0.35 – 2.42)
Jola	0.036	0.95^2^	1.04 (0.33 – 3.28)
Others	0.043	0.952	1.04 (0.26 – 4.21)
**Level of Education**			
Not educated	-0.030	0.908	0.97 (0.58 – 1.63)
Educated*			1
**Religion**			
Christianity	0.147	0.900	1.16 (0.12 – 11.50)
Islam*			1
**Employment** **status**			
Employed	-0.946	0.446	0.39 (0.03 – 4.42)
Not employed*			1
**Parity**			
Primipara	1.350	0.259	3.86 (0.37 – 40.33)
Multipara	0.103	0.744	1.11 (0.60 – 2.05)
Grandmultipara*			1
**Number of** **children**			
0	-1.768	0.208	0.17 (0.1 – 2.59)
1 – 4	-0.604	0.197	0.55 (0.22 – 1.37)
>4*			1
**Gestational Age**			
First trimester	-0.806	0.274	0.45 (0.11 – 1.90)
Second trimester	-0.889	0.216	0.41 (0.10 – 1.68)
Third trimester*			1
**Constant**	2.330		

R^2^ = 3.9% – 5.2%, Omnibus Tests of model coefficient = 11.11 (p=0.803)

* Reference category

Dependent variable was positive test results for STIs. The variables in the model explained 3.9% to 5.2% of the variation observed in the prevalence of STIs amongst the respondents. The model was statistically useful (Omnibus Tests of model coefficient = 11.11, p=0.803).

## Discussion

This study revealed a high prevalence of STIs among pregnant women attending ANC in the West Coast region of The Gambia. The isolated pathogenic agent includes *Treponema pallidum*, HIV, *Trichomonas vaginalis*, *Neisseria gonorrhoea, Chlamydia trachomatis, Candida albicans* and *Streptococcus agalactiae*.

This worrisome high prevalence of STIs among the pregnant women could probably be due to the low educational status of the women and the poor knowledge of STIs including HIV/AIDs and their symptomatology as reported among the same population in a previous study.^10^ It therefore means that many of these women could manifest symptoms of STIs without paying attention to it. This portends a great danger to the health of the women and that of their unborn child. A similar study carried out in Tanzania among pregnant adolescent girls attending ANC clinic also revealed a high prevalence of STIs.^11^ The education and empowerment of women is significant in the reduction of the high burden of STIs amongst women in the African region. The most prevalent pathogenic organism isolated in the pregnant women was *Candida albicans*. This finding is comparable to previous studies done in The Gambia^7^ and Nigeria^12^–^14^ where the prevalence of *Candida albicans* ranged from 21.1% to 60.8%. Although *Cadida species* are ubiquitous in nature, it can cause various infections from superficial to invasive forms. Pregnant women have a two-fold increase in the prevalence of vaginal colonization by *Candida* species compared to non-pregnant women. This association is influenced by increased level of circulating estrogens, and deposition of glycogen and other substrates in the vagina during pregnancy. Vaginal candidiasis in pregnant women has been reported to cause blood stream infections particularly in low birth weight and premature infants. Using molecular typing techniques, vertical transmission of *Candida albicans, Candida parapsilosis*, and *Candida glabrata* have been documented.^15^,^16^ Educating women on how to clean themselves after using the toilet (such as wiping from front to back to avoid spreading yeast from the anus to the vagina), early diagnosis and prompt treatment will reduce the spread of candidiasis.

GBS that commonly inhabits the genitals, lower urinary and gastrointestinal tracts of pregnant women have been shown to be a leading cause of perinatal bacterial infections including chorioamnionitis and endometritis in women, and pneumonia, septicemia and meningitis in the newborn including the increased risk of preterm delivery. The risk of neonatal infection is highest if mothers are colonized with GBS in the third trimester. The prevalence rate of GBS in this study was lower than what was reported in Eastern African countries of Uganda (28.8%),^17^ Ethiopia (20.9%),^18^ and Tanzania (23%).^19^ But a far lower prevalence of 1.8% was reported in Maputo, Mozambique.^20^ The administration of antibiotics to pregnant women identified with GBS during labour is a useful public health preventive measure against complications in both the woman and the baby. Data from developing countries confirm that maternal syphilis still remains an extremely important cause of perinatal morbidity and mortality.^2^ The natural history of syphilis acquired in pregnancy is believed to follow the sequential stages of primary, secondary, and latent syphilis that have been observed in untreated, non-pregnant adult cases. The consequences of untreated syphilis in pregnant women include: low birth weight, stillbirth, mid trimester spontaneous abortion, preterm birth, perinatal death, and congenital infection to the surviving infant.^21^–^23^ The prevalence rate of syphilis of 6.8% in this study is very worrisome even though it was lower than the 10% earlier reported in The Gambia in 2001.^8^ This calls for scaling up of intervention measures to reduce it to rates comparable to 2.5% reported in Tanzania in 2011,^24^ and even lower.

The prevalence rate of HIV in this study was higher than the estimates of 2.1% (for females) obtained from The Gambian Demographic and Health Survey (DHS) in 2013 and 1.4% from the HIV National Sentinel Surveillance (NSS) in 2014.^25^ The high proportion of pregnant women who were uneducated and the correspondingly high prevalence rate of STIs in this study is a probable reason for this finding. In addition, when disaggregated according to regions, the Brikama District where this study was carried out recorded the highest prevalence of 2.7% in the NSS data and came third with a prevalence of 2.6% in the DHS data. This calls for further investigations so that necessary steps can be taken to reverse the increasing trend HIV infection in Brikama District in particular and in The Gambia in general. Interventions targeted at behavioural change using the ABC approach (A-Abstinence, B-be faithful and C-Use condom), health education on the mode of transmission, encouraging HIV counselling, testing and treatment, and prevention of mother to child transmission (PMTCT) will be beneficial to pregnant women, their children, families, communities, and country at large.

In this study, it was quite encouraging that the prevalence of *Trichomonas vaginalis, Neisseria gonorrhoea* and *Chlamydia trachomatis* were generally low because of their association with the incidence of HIV infection.^26^ Trichomonas vaginalis, the causative agent of trichomoniasis, is a common cause of vaginitis and it has been associated with increased viral shedding and increased acquisition of HIV potentially contributing to a significant number of additional HIV infections globally.^27^ *Neisseria gonorrhea*, regarded as one of the most common bacterial causes of STI worldwide, is often asymptomatic in women yet it is associated with an increased risk of HIV transmission and it can readily be transmitted from mother to child during vaginal delivery.

## Limitation

It was a hospital based study carried out in only one region of The Gambia and therefore the results may not be generalized to all pregnant women in the entire country. However, the strength of this study lies on the fact that it serves as a source of current data for a multicentre study to further explore issues of STIs among pregnant women in the whole of The Gambia.

## Conclusion

We found a high prevalence of STIs among pregnant women attending ANC in the West Coast region of The Gambia. *Candida albicans* was the most prevalent pathogenic agent isolated while *Chlamydia trachomatis* was the least isolated. Public health intervention programmes should be strengthened to promote the sexual and reproductive health of pregnant women in The Gambia. These should include but not limited to the following: health education on the importance of personal hygiene, STIs, their causative agents as well as how to prevent them should be made a priority by health care workers during ANC visits by pregnant women. The spouses of pregnant women who test positive for STIs after routine screening during ANC visits should be treated to avoid continuous re-infection. Active search for symptoms of STIs such as vaginal discharge should be incorporated into the standard ANC package for pregnant women in The Gambia.

## References

[1] World Health Organization (2016–2021). Global Health Sector Strategy on Sexually Transmitted Infections.

[2] The World Bank Sexually Transmitted Infections in Developing Countries.

[3] World Health Organization (2009). Global Health Risks: Mortality and Burden of Disease Attributable to Selected Major Risks.

[4] World Health Organization (2019). Sexually Transmitted Infections (STI).

[5] National Institutes of Health (2017). How do sexually transmitted diseases and sexually transmitted infections (STDs/STIs) affect pregnancy?.

[6] Mullick S, Watson-Jones D, Beksinska M, Mabey D (2005). Sexually transmitted infections in pregnancy: prevalence, impact on pregnancy outcomes, and approach to treatment in developing countries. Sex Transm Infect.

[7] Mabey DC, Lloyd-Evans NE, Conteh S, Forsey T (1984). Sexually transmitted diseases among randomly selected attenders at an antenatal clinic in The Gambia. Br J Vener Dis.

[8] Shaw M, van der Sande M, West B, Paine K, Ceesay S, Bailey R (2001). Prevalence of herpes simplex type 2 and syphilis serology among young adults in a rural Gambian community. Sex Transm Infect.

[9] Mboto CI, Fielder M, Davies-Russell A, Jewell AP (2009). Prevalence of HIV-1, HIV-2, Hepatitis C and co-infection in The Gambia. West African Journal of Medicine.

[10] Baldeh A, Isara AR (2019). Knowledge of sexually transmitted infections amongst pregnant women attending antenatal clinics in West Coast Region of The Gambia. African Journal of Reproductive Health.

[11] Hokororo A, Kihunrwa A, Hoekstra P, Kalluvya SE, Changalucha JM, Fitzgerald DW (2015). High prevalence of sexually transmitted infections in pregnant adolescent girls in Tanzania: a multi-community cross-sectional study. Sex Transm Infect.

[12] Ekanem EI, Ekott M, Udo AE, Efiok EE, Inyang-Out A (2012). Prevalence of sexually transmitted diseases in pregnant women in Ikot Ekpene, a rural Community in Akwa Ibom State, Nigeria. Open Journal of Obstetrics and Gynecology.

[13] Usanga V, Abia-Bassey L, Inyang-etoh P, Udoh S, Ani F, Archibong E (2009). Prevalence of sexually transmitted diseases in pregnant and non-pregnant women in Calabar, Cross River State, Nigeria. The Internet Journal of Gynecology and Obstetrics.

[14] Nnadi DC, Singh S (2017). The Prevalence of genital Candida species among pregnant women attending antenatal clinic in a tertiary health center in North-west Nigeria. Sahel Medical Journal.

[15] Hay P, Czeizel AE (2007). Asymptomatic Trichomonas and Candida colonization and pregnancy outcome. Best Pract Res Clin Obstet Gynaecol.

[16] Bliss JM, Basavegowda KP, Watson WJ, Sheikh AU, Ryan RM (2008). Vertical and horizontal transmission of Candida albicans in very low birth weight infants using DNA fingerprinting techniques. Pediatr Infect Dis J.

[17] Namugongo A, Bazira J, Fajardot Y, Joseph N (2016). Group B Streptococcus Colonization among Pregnant Women Attending Antenatal Care at Tertiary Hospital in Rural Southwestern Uganda. International Journal of Microbiology.

[18] Mohammed M, Asrat D, Woldeamanuel Y, Demissie A (2012). Prevalence of GBS Colonization Among Pregnant Women Attending Antenatal Clinic of Hawassa Health Center, Hawassa, Ethiopia. Ethiop J Health Dev.

[19] Joachim A, Matee MI, Massawa FA, Lyamuya EF (2009). Maternal and Neonatal Colonization of group B streptococcus at Muhimbili National Hospital in Dar es Salaam, Tanzania: prevalence, risk factors and antimicrobial resistance. BMC Public Health.

[20] de Steenwinkel FD, Tak HV, Muller AE, Nouwen JL, Oostvogel PM, Mocumbi SM (2008). Low carriage rate of group B streptococcus in pregnant women in Maputo, Mozambique. Trop Med Int Health.

[21] American Pregnancy Association (2019). Syphilis during pregnancy.

[22] De Santis M, De Luca C, Mappa I, Spagnuolo T, Licameli A, Straface G, Scambia G (2012). Syphilis Infection during pregnancy: fetal risks and clinical management. Infectious Diseases in Obstetrics and Gynecology.

[23] Oswal S, Lyons G (2008). Syphilis in pregnancy. Contin Educ Anaesth Crit Care Pain.

[24] Manyahi J, Jullu BS, Abuya MI, Juma J, Ndayongeje J, Kilama B (2015). Prevalence of HIV and syphilis infections among pregnant women attending antenatal clinics in Tanzania, 2011. BMC Public Health.

[25] Republic of The Gambia (2015). The Gambia Global AIDS Response Progress Report Response.

[26] Kinuthia J, Drake AL, Matemo D, Richardson BA, Zeh C, Osborn L (2015). HIV Acquisition during pregnancy and postpartum is associated with genital infections and partnership characteristics: a cohort study. AIDS.

[27] McClelland RS, Sangare L, Hassan WM, Lavreys L, Mandaliya K, Kiarie J (2007). Infection with Trichomonas vaginalis Increases the Risk of HIV-1 Acquisition. The Journal of Infectious Diseases.

